# Laser peripheral iridotomy versus laser peripheral iridotomy plus laser peripheral iridoplasty in the treatment of multi-mechanism angle closure: study protocol for a randomized controlled trial

**DOI:** 10.1186/s13063-017-1860-4

**Published:** 2017-03-17

**Authors:** Shida Chen, Jianhua Lv, Sujie Fan, Hong Zhang, Lin Xie, Ling Xu, Bing Jiang, Huipin Yuan, Yuanbo Liang, Shuning Li, Pingyan Chen, Xiulan Zhang, Ningli Wang, for the Multi-mechanism Angle Closure Study (MACs) group

**Affiliations:** 1 Zhongshan Ophthalmic Center, State Key Laboratory of Ophthalmology, Sun Yat-sen University, 54 South Xianlie Road, Guangzhou, People’s Republic of China; 2 Hebei Provincial Eye Hospital, Xingtai City, Hebei People’s Republic of China; 3 Handan Eye Hospital, Handan City, Hebei People’s Republic of China; 4 Department of Ophthalmology, Tongji Hospital, Tongji Medical College, Huazhong University of Science and Technology, Wuhan, Hubei People’s Republic of China; 5 Department of Ophthalmology, Daping Hospital, The Third Military Medical University, Chongqing, Hubei People’s Republic of China; 6 Liaoning He University, He Eye Hospital, Shenyang, Liaoning People’s Republic of China; 7 Department of Ophthalmology, the Second Xiangya Hospital, Central South University, Changsha, Hunan People’s Republic of China; 8 Department of Ophthalmology, the Second Affiliated Hospital of Harbin Medical University, Harbin, Heilongjiang People’s Republic of China; 9 Clinical and Epidemiological Research Center, the Affiliated Eye Hospital of Wenzhou Medical University, Wenzhou, Zhejiang People’s Republic of China; 10 Beijing Tongren Eye Center, Beijing Tongren Hospital, Capital Medical University, Beijing Ophthalmology and Visual Science Key Laboratory, No. 1 Dong Jiao Min Xiang Street, Dongcheng District, Beijing, People’s Republic of China; 11 Department of Biostatistics, School of Public Health and Tropical Medicine, Southern Medical University, Guangzhou, Guangdong People’s Republic of China

**Keywords:** PAC, PACG, Primary angle closure, Laser peripheral iridoplasty, Laser peripheral iridotomy, LPI, LPIP, Primary angle-closure glaucoma

## Abstract

**Background:**

China has the largest burden of primary angle-closure glaucoma (PACG) worldwide. The mechanism of the angle closure is complex and includes pupillary block and non-pupillary block. Currently, opinion is that laser peripheral iridotomy (LPI) alone is not sufficient to prevent disease progression. Laser peripheral iridoplasty (LPIP) is an alternative and effective way of widening the angle recess in eyes that are affected by primary angle closure (PAC). However, it is not known if greater benefit would be achieved using LPI plus LPIP for PAC with multiple mechanisms (MAC). Thus, the aim of this study is to demonstrate if LPI plus LPIP would be more effective than single LPI in controlling the progression of PAC with multiple mechanisms, based on ultrasound biomicroscopy (UBM) classification. A secondary aim is to determine whether or not this would result in the use of less medication and/or prolong the time to antiglaucoma surgery.

**Methods:**

This multiple-mechanism angle-closure study will comprise a 3-year, multicenter, randomized, parallel-group, open-label, superiority trial, the aim of which will be to evaluate the safety and efficacy of LPI plus LPIP versus LPI for PAC. It is anticipated that 240 adults, diagnosed with PAC (the mechanism of angle closure will be assessed by UBM and it will be determined whether or not it involves multiple mechanisms) will be recruited from ten ophthalmic centers in China. Participants will be randomly allocated to receive either single LPI or LPI plus LPIP. Participant assessment will be designed to test the rate of disease progression and who will be followed up for 3 years. The primary outcome will be the disease progression rate and a comparison will be made between the LPI and LPI plus LPIP groups using Pearson’s χ^2^ test. Logistic regression analysis will be performed to account for the central effect.

**Discussion:**

If the LPI plus LPIP is found to significantly decrease the rate of PAC progression, this intervention could potentially be a standard therapy to be used to treat PAC when multiple mechanisms are involved in angle closure. Subsequently, this would have the potential to delay the rate of PAC progression to PACG and delay the time to the administration of antiglaucoma medication or trabeculectomy surgery.

**Trial registration:**

ClinicalTrials.gov, NCT02613013. Registered on 24 November 2015.

In fact, the study was due to start in late October 2015, however, there were no patients recruited in October, and when we registered at ClinicalTrials.gov on 5 November 2015, we received suggestions on the English translation of our protocol from the PRS Team at Clinicaltrial.gov, so the final successful registration date was on 24 November 2015.

**Electronic supplementary material:**

The online version of this article (doi:10.1186/s13063-017-1860-4) contains supplementary material, which is available to authorized users.

## Background

Glaucoma is the second leading cause of irreversible blindness and it is estimated that there will be 79.6 million glaucoma patients globally by 2020. Although angle-closure glaucoma (ACG) only accounts for 26% of all glaucoma, it is expected that angle-closure glaucoma will affect 87% of Asian glaucoma patients, with bilateral blindness recorded in 5.9 million people with ACG [1]. It was found in a population-based study that China has the largest burden of primary angle-closure glaucoma (PACG) in the world [2].

Laser peripheral iridotomy (LPI) is the suggested initial treatment for primary angle closure (PAC) and early PACG as it eliminates pupillary block, allowing the convex iris to flatten, while widening the anterior chamber angle [3]. However, the pathophysiological features that underlie PAC are complex and include pupillary block and non-pupillary block mechanisms [4].

It was found in our previous ultrasound biomicroscopy (UBM)-based study that multiple mechanisms of angle closure were found in approximately 55% of PACG patients. The causes of the angle closure include pupillary block leading to peripheral iris bombe, anterior rotation of the ciliary body, and a thickened and anteriorly located peripheral iris [5]. Therefore, LPI alone may not be effective in angle closure caused by multiple mechanisms.

It was discovered in the Liwan Eye Study and Zhongshan Angle-Closure Prevention Trial that approximately 20% of Chinese eyes with suspected PAC had residual angle closure after single iridotomy and the greater angle width following iridotomy further decreased as time went on [6, 7]. Choi et al*.* followed up eyes with peripheral anterior synechia (PAS) after LPI for a mean of 34.4 months and found that disease progressed in 32% of the eyes due to the existence of a non-pupillary block component in angle closure, i.e., plateau iris [8]. It was shown in a 2-year follow-up study on primary angle closure suspects (PACS) after LPI that roughly 28% of the patients progressed to PAC. A decreasing anterior chamber area was predictive of progression from PACS to PAC [9].

Contraction burns to the peripheral iris result from peripheral iridolasty, leading to contraction of the peripheral iris stroma and the creation of a space between the anterior iris surface and the trabecular meshwork [10]. It has also been suggested that this effectively opens up the appositionally closed portions of the drainage angle, even resulting in a synechiolysis effect [11]. Ritch et al*.* found that laser peripheral iridoplasty (LPIP) was highly efficient in eliminating residual appositional closure following LPI caused by plateau iris [12].

Therefore, it is reasonable to hypothesize that LPI plus LPIP would be more efficacious in opening up the angle closure caused by multiple mechanisms and would provide greater intraocular pressure (IOP) control. It was found in our previous randomized controlled trial that LPI plus LPIP resulted in superior reduction in the PAS surface area than LPI alone in synechial PAC and PACG after 1-year follow-up, although a similar magnitude of IOP reduction was reported for the two interventions [13]. However, to our knowledge, a guideline has not been published, nor have any studies been conducted, on the treatment of PAC based on the clinical research associate classification of various mechanisms of angle closure.

## Methods

### Study objective

The aim of this study is to demonstrate the efficacy of LPI plus LPIP versus LPI in reducing the rate of 3-year disease progression in patients with multiple mechanisms of angle closure based on UBM classification. A secondary objective is to assess the safety of LPI plus LPIP in the study population over its duration.

### Trial design

This study has been designed as a 3-year, multicenter, randomized, parallel group, open-label, superiority trial. Interim analysis will not take place during this study. A completed Standard Protocol Items: Recommendations for Interventional Trials (SPIRIT) checklist for the trial is available (see Additional file 1).

### Participant characteristics

Patients from the ten ophthalmic centers in China will be assessed for suitability regarding participation in the study by the primary investigator at each center and by the researchers who confirm the UBM reading results, using the criteria detailed herein.

### Inclusion criteria

Inclusion criteria are as follows:

- Patients with a clinical diagnosis of PAC, with IOP ≤ 30 mmHg and PAS ≤ 270 °.
- Patients with PAC with multiple mechanisms based on the results of a UBM examination. (“Multiple mechanisms” is defined as PAC caused by pupillary block plus at least one non-pupillary block mechanism).
- Patients with visual acuity ≥ 20/40.
- Patients who are aged 40–75 years and of Chinese descent.

If both eyes of the patient are eligible for inclusion in the study, the one with greater diminished visual acuity will be selected. Only one eye per patient will be eligible for inclusion in the study.

Eligible patients who are already on antiglaucoma medication will be required to undergo a polypharmacy washout before the randomization process is applied. Different washout periods apply to the various medications, for example, prostaglandin analogs are eliminated after 4 weeks, beta blockers after 3 weeks, adrenergic agonists after 2 weeks, and cholinergic agonists and carbonic anhydrase inhibitors after 5 days. The polypharmacy washout process will be stopped in patients with an IOP > 30 mmHg during the washout period and they will be withdrawn from the study.

A diagnosis of PAC will be based on the 2010 Glaucoma Preferred Practice Pattern® of eyes that are classified as having at least 180 ° of iridotrabecular contact and an elevated IOP (IOP ≥ 21 mmHg) on at least one occasion, with this result recurring three times, or PAS. Visual field loss and glaucomatous optic neuropathy, as defined herein, are exclusion criteria.

### Exclusion criteria

The exclusion criteria are as follows:

- Patients who are unwilling or unable to provide consent to participation in the study, who are unwilling to submit to the randomization process, or are unable to return for the scheduled protocol visits.
- Patients with angle closure due to secondary causes (i.e., subluxated lens, neovascular, uveitic, traumatic and postoperative).
- Patients who have undergone previous incisional intraocular surgery or ocular laser in the study eye (LPI, LPIP, cyclodestructive procedure, and cataract surgery).
- Patients with PAC with glaucomatous neuropathy.
- Patients who have a cataract in the study eye and who anticipate having cataract surgery in the next 3 years. (The existing cataract could affect the visual field and fundus examination and it is likely that visual acuity would be < 20/40 due to the existing cataract).
- Patients using IOP-lowing drugs who do not agree to submit to the medication washout process.
- Patients who require glaucoma surgery combined with other ocular procedures, such as cataract surgery, penetrating keratoplasty, and retinal surgery), or who anticipate the need for urgent additional ocular surgery.
- Patients with other coexisting ocular diseases, such as an abnormal cornea or a corneal infection, iridocorneal endothelial syndrome, anterior segment dysgenesis, nanophthalmos, high myopia (>6.0 D), chronic or recurrent uveitis, ocular cancer, trauma, central retinal vein occlusion, central retinal artery occlusion, and retinal detachment).
- Patients with a corneal endothelium count of < 1000/mm^2^.
- Patients who require the long-term use of local or systemic steroids.
- Patients who are unwilling to discontinue contact lens use after surgery.
- Patients who are participating in other clinical trials.
- Pregnant or nursing women.
- Patients with severe systemic disease, such as diabetes mellitus, hypertension, end-stage cardiac disease, nephropathy, respiratory disease, and cancer.
- Patients who are allergic to pilocarpine or alcaine.
- Patients with a contraindication to laser treatment for ocular disease.

### Recruitment procedure

**Fig. 1:**
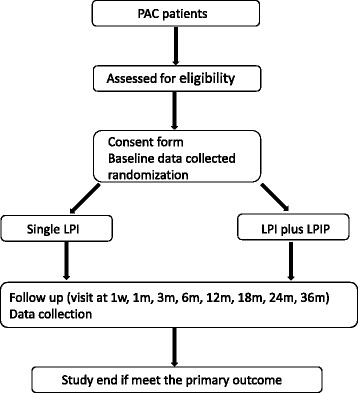
Participant flow diagram. *LPI* laser peripheral iridotomy, *LPIP* laser peripheral iridoplasty, *PAC* primary angle closure

The participant flow diagram is showed in Fig. 1.

### Sample size

The sample size calculations are based on published PAC disease progression with LPI and LPIP treatment. Disease progression is defined as glaucomatous optic nerve injury, increased IOP for which antiglaucoma drugs are required, and PAS progression according to the published clinical trials [13–15]. It is assumed that the 3-year PAC progression rate after receiving LPI will be approximately 55%, and the rate for LPI plus LPIP will be 35%, with 80% statistical power and a two-sided test at 5% significance. Thus, allowing for 20% loss to follow-up, a sample size of 240 participants for this study is required (calculated using nQuery Advisor® + nTerim® 3.0 software; Statsols, Boston, MA, USA). There will be 120 patients in each treatment arm. The anticipated number of patients in the study (240) will be recruited over a 12-month period.

### Randomization process

Patients will be randomly assigned to treatment with either single LPI or LPI plus LPIP using a 1:1 ratio via an online central randomization system. (This system is run by personnel in the Department of Biostatistics, Southern Medical University, Guangzhou, China), using the IOP as the stratification parameter. Patients will be stratified into a high IOP subgroup (21 mmHg to ≤ 30 mmHg) and a low IOP subgroup (≤21 mmHg) since it is likely that IOP will vary in the enrolled PAC participants. This procedure will ensure that there is a nearly equal number of patients in each treatment group early on in the trial and that the investigator will not be able to predict the next treatment assignment. In addition, the number of patients in the high and low IOP arms will almost be equal. The intervention date is the study entry date. Dates for all postoperative follow-up visits will be computed from this date.

### Blinding

This study is an open-label trial.

### The classification of ultrasound biomicroscopy-based angle closure

Patients who have been diagnosed with PAC will be invited to undergo a UBM examination (SW3200L®; Suowei Electronic Company, Tianjin, China). The UBM examination will be conducted at every center by a technician with at least 3 years’ experience in operating a UBM machine and who has also received training according to the standard operating procedures of the study. The UBM examination will be performed in a dark room with illumination of ≤ 5 Lux. Images of the four quadrants (the superior, inferior, nasal, and temporal quadrants) and one image of the central anterior chamber will be acquired in supine subjects.

The criteria for acceptable images will be clear visualization of the scleral spur, angle, ciliary body, and the half chord of the iris. After obtaining the UBM results, the research physicians will upload the UBM images (containing anonymized information) to an online computer system that is only accessible to the UBM analysts, who will send the classification results on to the research physician after reviewing the images and determining the results according to the UBM classification criteria. The UBM analysts will consist of three glaucoma specialists. Every UBM figure will be read by at least two specialists. If agreement on the results cannot be reached by the two specialists, a third will be approached in order to obtain consensus.

**Fig. 2:**
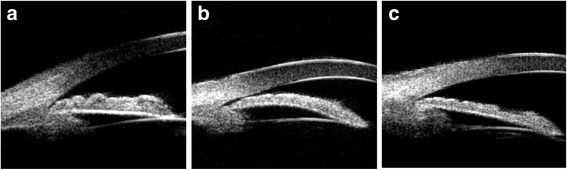
UBM-based angle closure configuration classification. **a** Thickening of the peripheral iris; **b** iris bombe; **c** anterior rotation of the ciliary body. *UBM* ultrasound biomicroscopy
- *Thickness of the peripheral iris:* the thickness of the peripheral iris will be graded with reference to limbal corneal thickness and is defined as greater than half the thickness of the limbal zone of the cornea (Fig. 2b).
- *Anterior rotation of the ciliary body:* the anterior rotation of the ciliary body will be judged according to the direction of the axis of the ciliary body in the anterior position, with the disappearance of the ciliary groove (Fig. 2c).

The UBM classification criteria are as follows [5, 16]:

- *Iris convexity:* judged according to the curvature of the posterior surface of the iris, the iris bombe is defined as the location of the posterior surface of the iris above a line drawn from the iris root to the iris margin of the pupil (Fig. 2a).

Thus, there will be seven configurations (combinations) of angle closure, as defined according to the aforementioned criteria, including:

- The iris bombe.
- Thickening of the peripheral iris.
- The anterior rotation of the ciliary body.
- The iris bombe plus thickening of the peripheral iris.
- The iris bombe plus the anterior rotation of the ciliary body.
- Thickening of the peripheral iris plus the anterior rotation of the ciliary body.
- The iris bombe plus thickening of the peripheral iris, plus the anterior rotation of the ciliary body.

Multiple mechanisms of angle closure are defined as combinations of two or three factors including the iris bombe, thickening of the peripheral iris, and the anterior rotation of the ciliary body.

Regarding the quadrants, the configuration of angle closure in one eye is defined as at least two quadrants belonging to one configuration; if two quadrants belong to one kind of configuration, and the other two belong to another kind of configuration, thereafter, reference will be made to the contralateral eyes.

### Intervention description

#### Single laser peripheral iridotomy

LPI will be performed using a VISULAS® 532 s diode laser (Carl Zeiss Meditec, Dublin, USA). Thirty minutes prior to the procedure, a drop of 2% pilocarpine will be instilled into the eye every 15 minutes. Topical anesthesia will be administered. The selected treatment site will be the superior nasal iris or iris crypt, where present. Treatment will be initiated with a pulse of 3–5 mJ. The power will be increased until patency is achieved and the opening of the iris is > 0.1 mm. Patency will be determined by direct visualization of the posterior chamber.

#### Laser peripheral iridoplasty plus laser peripheral iridotomy

LPIP will be applied using a VISULAS® 532 s diode laser (Carl Zeiss Meditec). Thirty minutes prior to the procedure, a drop of 2% pilocarpine will be instilled into the eye every 15 minutes, Topical anesthesia will be administered. Twenty to 30 ms spots of 250–300 mW power, measuring 300–500 microns, will be applied for a duration of 400–500 ms. Power will be modified arbitrarily until an effective iris contraction is obtained. Effective iris contraction will be considered to constitute concentric movement around the laser spot, with minimal iris pigmentation and immediate angle opening, as observed through a Goldmann® lens mirror. Power will be lowered if a bursting sound is perceived, or in the event of pigment dispersion, air bubbles, or considerable pain. LPI will be performed after the LPIP procedure is complete.

### Primary outcome measures

The PAC progression rate will be determined by the number of patients in whom the disease progresses following laser treatment in each group within the 3-year follow-up period. PAC progression is defined as the presence of any of the following:

- Acute angle-closure crisis.
- An IOP measurement taken 1 month after the laser procedure that is 8 mmHg higher than that recorded at treatment initiation.
- An IOP measurement taken 1 month after the laser procedure that is ≥ 22 mmHg when measured three times in succession.
- The progression of PAS ≥ 1 clock hour within 3 years of the laser procedure, as determined by gonioscopy.
- Glaucomatous neuropathy within 3 years of the laser procedure.

Acute angle-closure crisis is defined as:

- The presence of at least two of the following symptoms: ocular or periocular pain, nausea and vomiting, blurred vision, and multicolored halos around lights (an occasional symptom).
- An IOP measurement of at least 22 mmHg, as measured by Goldmann® applanation tonometry.
- The presence of at least three of the following signs: conjunctival injection, corneal edema, a mid-dilated unreactive pupil, and a shallow anterior chamber.

Glaucomatous neuropathy is defined as:

- The documented progression of diffuse thinning and focal narrowing or notching of the optic disc rim.
- A cup disc ratio of ≥ 0.6.
- A cup-disc asymmetry ratio of ≥ 0.2 for similar-sized eyes or optical discs.
- Diffuse or localized abnormalities in the peripapillary retinal nerve fiber layer.
- The presence of glaucomatous visual field defect, defined as reproducible visual field defects, including nasal step, arcuate field defect, or paracentral depression in the clusters of the test sites.
- Visual field loss in the upper hemifield that is different to that in the lower hemifield.
- The absence of any other known explanation.

### Secondary outcome measures

Secondary outcome measures include the following:

- Determining, through the administration of a questionnaire, whether or not additional medication (to control IOP) or additional surgery is required to control the progression of PAC.
- Establishing whether or not there are any changes in the best-corrected visual acuity after the laser procedure.
- Determining the number of corneal endothelium cells.
- Establishing any change in the angle width and configuration, as measured by UBM.

### Safety evaluation

An evaluation of the safety of the procedures, including any laser-related complications and adverse events, will be conducted and will cover:

- *Corneal injury:* corneal injury will be sought via an examination by slit lamp and by conducting a corneal endothelium cell count.
- *Anterior uveitis:* inflammation in the anterior chamber is indicative of anterior uveitis.
- *Changes in the pupil:* any changes to the pupil will be established following an examination by slit lamp.
- *Lens injury:* lens injury will be determined following an examination by slit lamp.
- *Retinal injury:* a fundus examination will be conducted to evaluate whether or not retinal injury has occurred.
- *Blindness:* blindness will constitute a major adverse event.

### Data collection and monitoring

All data will be collected at the scheduled follow-up times (Table 1). Trained clinical research associates at each center will examine the data on the patient information sheet, all of which will be entered into a trial-specific online database, Research Electronic Data Capture®. The data manager will monitor the data in a timely manner to control the quality of the data. Access to the final dataset will be limited to the trial administrator and the statistician.

**Table 1:** Schedule of visits and examination items

Visit number	1	2	3	4	5	6	7	8	9	10	11	12
Examination item	-7 to -1 day	Laser day	1 W (±1 D)	1 M (±1 W)	3 M (±1 W)	6 M (±1 W)	1 Y (±2 W)	1½ Y (±2 W)	2 Y (±2 W)	3 Y (±2 W)	4 Y (±2 W)	5 Y (±2 W) EOS
Informed consent	X											
Demographics data	**X**											
Medical history	**X**											
Vital signs	**X**	**X**										X
Height and weight	**X**											
Medication record	**X**		X	X	X	X	X	X	X	X	X	X
Refraction	**X** ^**b**^			X		X	X	X	X	X	X	X^**b**^
Visual acuity	**X** ^**b**^		X	X	X	X	X	X	X	X	X	X^**b**^
Slit lamp biomicroscopy	**X** ^**b**^		X	X	X	X	X	X	X	X	X	**X** ^**b**^
Fundus examination	**X** ^**b**^			X		X	X	X	X	X	X	**X** ^**b**^
IOP (Goldmann)	**X** ^**b**^	**X** ^**b**^	**X** ^**b**^	**X** ^**b**^	**X** ^**b**^	**X** ^**b**^	**X** ^**b**^	**X** ^**b**^	**X** ^**b**^	**X** ^**b**^	**X** ^**b**^	**X** ^**b**^
A scan	**X** ^**b**^						X		X	X	X	**X** ^**b**^
Endothelial cell count	**X** ^**b**^			X		X	X		X	X	X	**X** ^**b**^
UBM	**X** ^**b**^			X		X	X	X	X	X	X	**X** ^**b**^
AS-OCT	**(X** ^**b**^ **)**			(X)		(X)	(X)	(X)	(X)	(X)	(X)	**(X** ^**b**^ **)**
Humphrey 24-2	**X** ^**b**^				X		X		X	X	X	**X** ^**b**^
Antiglaucoma surgery				X	X	X	X	X	X	X	X	X
Laser treatment		X										
Adverse event	**X**	**X**	**X**	**X**	**X**	**X**	**X**	**X**	**X**	**X**	**X**	**X**

X^b^: examination item for both eyes; (x): optional examination item (AS-OCT)

*EOS*, the end of the study, *IOP* intraocular pressure, *UBM* ultrasound biomicroscopy, *AS-OCT* anterior segment optical coherence tomography, *W* week, *M* ﻿month, *Y* year

### Data analysis

The statistical tests will be two-sided. A level of 0.050 will constitute statistical significance. A comparison of the normally distributed continuous variables between two groups, expressed as mean ± standard deviation, will be determined using two independent *t* tests. The natural logarithmic transformation will be applied to variables that are positively skewed. Non-normally distributed continuous variables, presented as the median and interquartile range, will be analyzed using the Wilcoxon rank-sum test. The ordinary variables, also presented as median and interquartile range or percentages, will be analyzed using Wilcoxon signed-rank test. Pearson’s chi-square test or Fisher’s exact test will be used, as appropriate, for the categorical data and expressed as percentages. If the primary outcome is not realized, the last observation carried forward method will be used to impute the reason why. Any other values will remain as absent. The baseline characteristics will be evaluated using intent-to-treat (ITT) analysis. Efficacy analysis will be applied to the ITT and per protocol populations. Reasons for loss to follow-up will also be presented.

An evaluation of the rate of disease progression after 3 years of follow-up will be conducted using efficacy analysis as the primary outcome measure. A comparison in this regard will be made between the LPI and LPI plus LPIP groups using Pearson’s χ^2^ test. Logistic regression analysis will be performed to evaluate the central effect and the progression rate adjusted to account for it.

The safety evaluation data, as per the secondary outcome, will be analyzed using a generalized linear model in consideration of any covariates or the central effect. Sensitivity analysis will be conducted to assess for heterogeneity. Statistical Analysis Software® 9.4 (SAS Institute Inc., Cary, NC, USA) will be used to analyze the collected data. The data analysis will be performed by personnel in the Department of Biostatistics, Southern Medical University, Guangzhou, China.

## Discussion

LPI alone is not considered to be sufficient to treat the large number of PAC patients with multiple mechanisms of angle closure in China. Laser treatment, based on the type of angle closure, is reasonable and necessary. A multicenter, prospective, randomized controlled trial on the optimal laser treatment for PAC is mandatory but is currently lacking. This trial is the first multicenter, prospective, randomized controlled trial that will evaluate the effect and safety of LPI plus LPIL, compared with LPI alone, for the treatment of PAC with multiple mechanisms based on UBM classification. The findings are expected to provide evidence that laser treatment for PAC should be based on the mechanism of angle closure.

Strengths of this trial include the fact that UBM-based angle-closure classification will be used, central randomization will be applied, the IOP will be classified as high and low, and the follow-up will be over a long term (3 years).

It was found by Jiang et al*.* that a thick peripheral iris was more relevant to a narrow angle configuration, based on UBM images in Chinese patients, although the anterior rotation of the ciliary body and the iris insertion was similar to that in narrow- and wide-angle eyes. However, on the other hand, it was demonstrated that multiple mechanisms were involved in angle closure in Chinese PAC patients [16].

Thus, the use of laser treatment (LPI plus LPIP), based on the UBM classification in the current study, was prudent. Though our previous randomized controlled trial stated that the use of LPI plus PLPI achieved an equivalent reduction in IOP in PAC and PACG, in that study both PAC and PACG patients with PAS were included and the type of angle closure was not classified, which may have influenced the results [13].

The classification of angle closure, based on the UBM images, is an issue that requires attention. Since there is no unified guideline on the UBM classification of angle closure, we referred to the previous hospital-based population study and the Liwan Eye Study carried out at Zhongshan Ophthalmic Center [5, 16]. Since UBM classification is semiquantitative, we used UBM analysts to avoid possible bias.

### Trial status

Recruitment for the MAC study began in November 2015. It is ongoing. Currently, more than 25% of the sample size has been attained. Since the recruitment rate is relatively slow, it is anticipated that the study will reach the recruitment target of 240 participants by June 2017.

## Additional files

Additional file 1: SPIRIT checklist. DOC 123 kb
Additional file 2: Reference number of all ethical bodies. DOCX 18 kb

## Abbreviations

IOP: Intraocular pressure
ITT: intent-to-treat
LPI: Laser peripheral iridotomy
LPIP: Laser peripheral iridoplasty
MAC: Multi-mechanism angle closure
PAC: Primary angle closure
PACG: Primary angle closure glaucoma
PACS: Primary angle closure suspect
PAS: Peripheral anterior synechia
UBM: Ultrasound biomicroscopy
