# Supplementary material for: Laser peripheral iridotomy versus laser peripheral iridotomy plus laser peripheral iridoplasty in the treatment of multi-mechanism angle closure: study protocol for a randomized controlled trial
Source: Trials. 2017 Mar 17;18:130. doi: 10.1186/s13063-017-1860-4 (PMC5356270; doi:10.1186/s13063-017-1860-4)
Supplement: Additional file 2: — Reference number of all ethical bodies. DOCX 18 kb [file 13063_2017_1860_MOESM2_ESM.docx]

| **PI** | **Centers** | **Ethical approval** |
| --- | --- | --- |
| Ningli Wang | Beijing Tongren Eye Center, Beijing Tongren Hospital, Capital Medical University | Ethics Committee of BeiJing Tongren Hospital, Capital Medical University (2016-0910) |
| Xiulan Zhang | Zhongshan Ophthalmic Center, State Key Laboratory of Ophthalmology, Sun Yat-Sen University | Ethics Committee of ZhongShan Ophthalmic Center，Sun Yat-sen University (2015MEKY058) |
| Yuanbo Liang | the Affiliated Eye Hospital of Wenzhou Medical University | Ethics Committee of the eye Hospital of Wenzhou Medical University  (KYK【2015】32) |
| Huipin Yuan | the Second Affiliated Hospital of Harbin Medical University | Ethics Committee of The second Affiliated Hospital of Harbin Medical University (2015- 218) |
| Hong Zhang | Tongji Hospital, Tongji Medical College, Huazhong University of Science and Technology | Ethics Committee of Tongji Hospital Tongji Medical College，Huazhong University of Science and Technology (TJ-C20150902) |
| Sujie Fan | Handan Eye Hospital | Ethics Committee of The Third Hospital of Handan City Eye Hospital (【2015】3) |
| Lin Xie | DaPing Hospital, The third military medical university | Ethics Committee of Daping Hospital，Research Institute of Surgery ,The Third Military Medical University （2015-18） |
| Bing Jiang | the second Xiangya hospital, central south university | Ethics Committee of The 2nd Xiangya Hospital of Central South University (2016-001) |
| Lin Xu | Liaoning He University, He Eye Hospital | Ethics Committee of He Eye Hospital (IRB（2015）K001.01) |
| Jianhua Lv | Hebei Provincial Eye Hospital | Ethics Committee of Hebei Provincial Eye Hospital (2015001) |
